# Characterization of the microtranscriptome of macrophages infected with virulent, attenuated and saprophyte strains of *Leptospira* spp.

**DOI:** 10.1371/journal.pntd.0006621

**Published:** 2018-07-06

**Authors:** Leandro Encarnação Garcia, Erivelto Corrêa de Araújo Junior, Larissa Martins Melo, Jaqueline Poleto Bragato, Juliana Regina Peiró, Valéria Marçal Félix de Lima, Márcia Marinho, Daniel Robert Arnold, Flavia Lombardi Lopes

**Affiliations:** 1 Department of Support, Production and Animal Health, São Paulo State University (Unesp), School of Veterinary Medicine, Araçatuba, SP, Brazil; 2 Department of Clinic, Surgery and Animal Reproduction, São Paulo State University (Unesp), School of Veterinary Medicine, Araçatuba, SP, Brazil; Insitut Pasteur de Tunis, TUNISIA

## Abstract

Leptospirosis is a bacterial zoonosis, caused by *Leptospira* spp., that leads to significant morbidity and mortality worldwide. Despite considerable advances, much is yet to be discovered about disease pathogenicity. The influence of epigenetic mechanisms, particularly RNA-mediated post-transcriptional regulation of host immune response has been described following a variety of bacterial infections. The current study examined the microtranscriptome of macrophages J774A.1 following an 8h infection with virulent, attenuated and saprophyte strains of *Leptospira*. Microarray analysis revealed that 29 miRNAs were misregulated following leptospiral infection compared to control macrophages in a strain and virulence-specific manner. Pathway analysis for targets of these differentially expressed miRNAs suggests that several processes involved in immune response could be regulated by miRNAs. Our data provides the first evidence that host miRNAs are regulated by *Leptospira* infection in macrophages. A number of the identified miRNA targets participate in key immune response processes. We suggest that post-transcriptional regulation by miRNAs may play a role in host response to infection in leptospirosis.

## Author summary

Leptospirosis is a zoonotic disease, distributed worldwide, affecting millions of people each year, and leading to sixty thousand deaths per year. These bacteria are found in soil and water and are eliminated by the urine of rodents, their natural reservoir. Through skin contact, bacteria can be acquired, infecting the host. Infection process in leptospirosis is not completely understood and here we add another layer of disease regulation. Recent studies have shown that pathogens can modulate host response. Our current study examined the expression of microRNAs in murine macrophages following an 8h infection with virulent, attenuated and saprophyte strains of *Leptospira*. This study provides the first evidence that these post-transcriptional regulatory molecules, microRNAs, are modulated in macrophages in a species and virulence-specific manner, following infection with different strains of *Leptospira* spp. These microRNAs are involved in the regulation of inflammatory and antimicrobial responses in the host and could lead to the identification of biomarkers or therapeutic targets for this disease.

## Introduction

Leptospirosis is a zoonosis of global importance, particularly in developing countries with tropical climates [1], and is caused by a highly invasive gram-negative spirochete known as *Leptospira*. This genus is comprised of 12 species and 250 serotypes between pathogenic and non-pathogenic strains [2–3], with *Leptospira interrogans* being the most common pathogenic species. Mortality rate is around 60,000 deaths per year and the annual number of severe cases can reach 1 million, placing leptospirosis as a major player in morbidity, and number of deaths, by zoonotic causes [4–5].

Rodents are natural reservoirs for these bacteria and they shed the pathogen in their urine, contaminating water and soil in urban and rural environments. Humans can be infected by skin contact, mainly in areas lacking sanitation [6]. During early infection, antibiotics are effective, however most vaccines available for veterinary application provide limited protection against more than 250 pathogenic *Leptospira* serovars [7–8]. Advances in research have been made through the use of conserved leptospiral proteins, aiming at better vaccine candidates for leptospirosis [9].

Macrophages play a central role in leptospirosis by phagocytizing bacteria in humans and other mammals [10–11]. Cinco *et al*. [12] suggest that *Leptospira* inside macrophages are fully capable of replication, and Li *et al*. [13] showed that *Leptospira* are capable of escaping host defense responses, however only in human macrophages.

*Leptospira interrogans* has higher pathogenicity due to components such as lipopolysaccharides, peptideoglycans, lipoproteins, glycoproteins and membrane proteins, which induce a robust inflammatory response [14–17]. Activation of innate immunity by toll-like receptors (TLRs 2/4) in macrophages is essential for host defense [18]. TLR activation and MyD88 recruitment, signaling through several pathways like mitogen-activated protein (MAP) kinases, NF-κB and pro-inflammatory cytokines, lead to B and T cell activation [19–21]. Another rapid response induced by *L*. *interrogans* is apoptosis in macrophages and hepatocytes. This pathway is activated by caspase 3 and 6 through a FADD–caspase-8-dependent pathway [22] involving intracellular free calcium ion (Ca2+) [23].

It is known that the pathogen/host interaction can significantly modify gene expression profiles in an infected host, and it was suggested that post-transcriptional regulation might be acting in this interaction [24]. MicroRNAs (miRNAs) are small non-coding RNAs spanning 20–22 nucleotides, and have a major role in posttranscriptional regulation of gene expression. These small RNAs negatively regulate protein synthesis through base pairing with partially complementary sequences in the 3´UTR region of target mRNAs, favoring their degradation or translational repression [25–27]. Each miRNA has the potential to target, thus controlling, hundreds of genes [28]. An extensive body of research indicates that several pathogens (viruses, parasites and bacteria) can affect miRNA expression in host cells [29–31]. Further, miRNAs associated with disease can act as biomarkers or therapeutic targets [32–33].

For this reason, miRNAs and their targets, modulated by the pathogen, are of great importance to comprehend the pathophysiology of leptospirosis. Based on the identification of targets of differentially expressed miRNAs, following infection of murine macrophages with different types of *Leptospira*, we report several canonical pathways that could be affected by infection. We have established, for the first time, that modulation of miRNAs is present in *Leptospira* infection, and obtained potential miRNA signatures for different strains, varying in virulence. We suggest that posttranscriptional regulation by miRNAs may play a role in host response to infection in leptospirosis. These findings add to the growing list of infectious diseases that involve miRNAs regulation by the host.

## Results

### Expression profile of miRNAs in macrophages infected with saprophyte, attenuated and virulent strains of *Leptospira* spp.

**Fig 1:**
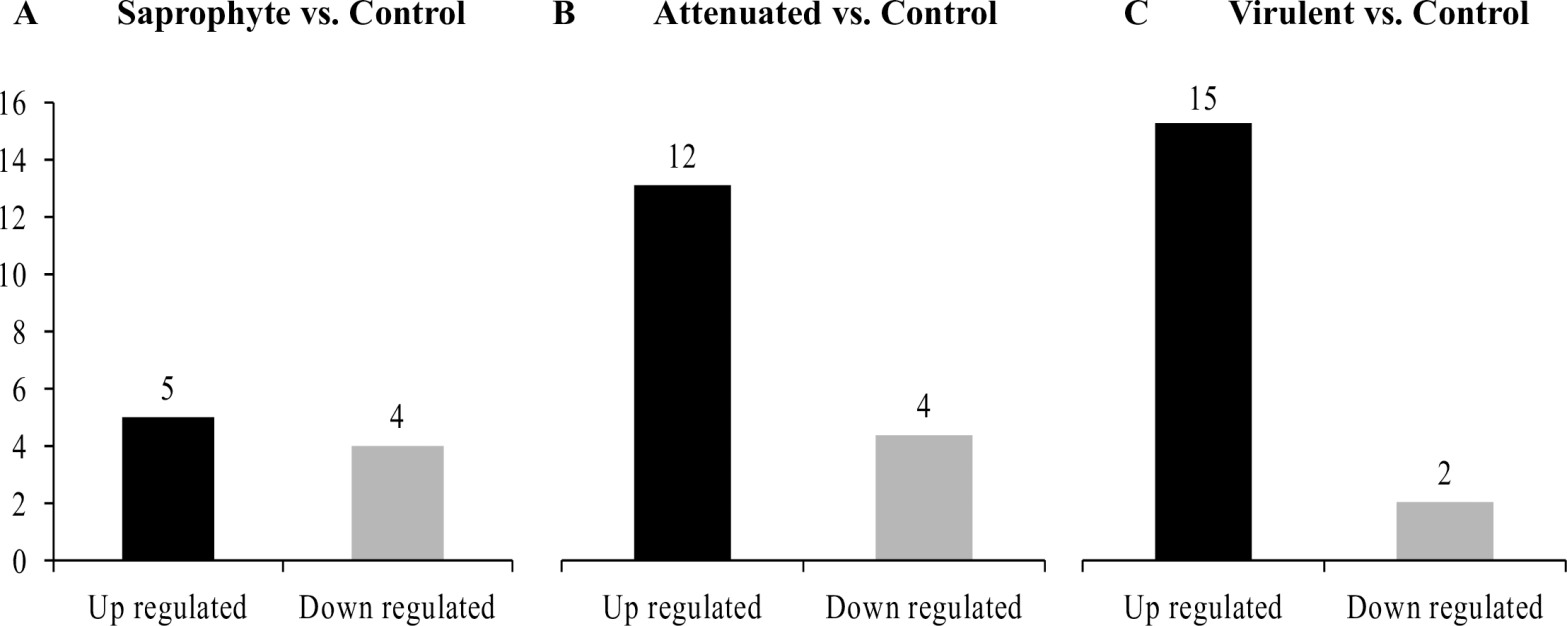
Quantity of miRNAs modulated by macrophages following 8h of infection by different strains of *Leptospira* spp. Total number of miRNAs/treatment **(n = 3/treatment; p-value<0.01; linear fold change ± 1.5) in the contrasts A) Saprophyte; B) Attenuated and C) Virulent vs. Non-infected Control**.

**Fig 2:**
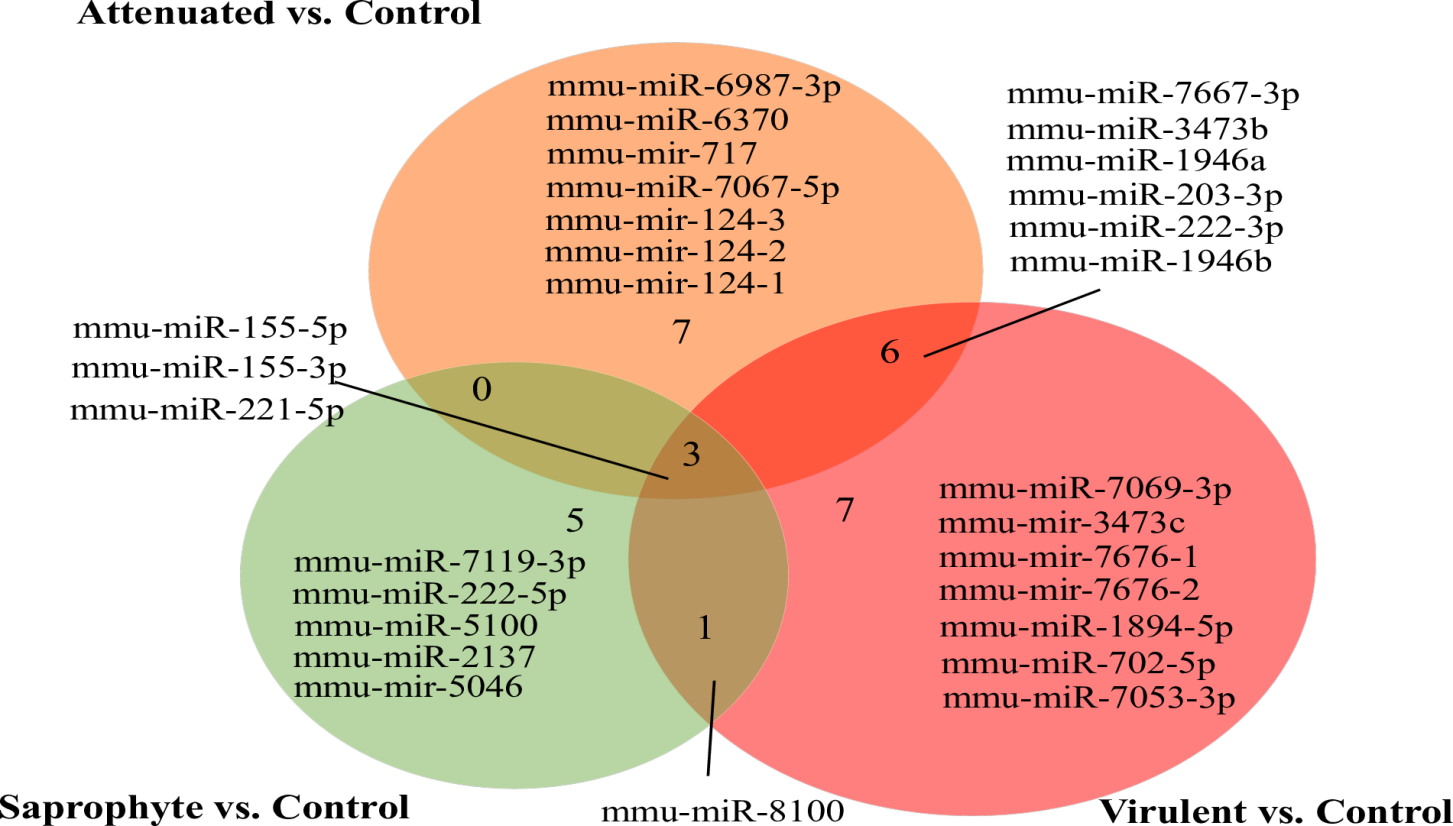
Venn diagram of miRNAs modulated by macrophages at 8h of infection by different strains of *Leptospira* spp. Total number of miRNAs/treatment **(n = 3/treatment; p-value <0.01; linear fold change ±1.5) in the contrasts Infected (Saprophyte; Attenuated and Virulent) vs. Non-infected Control**.

**Fig 3:**
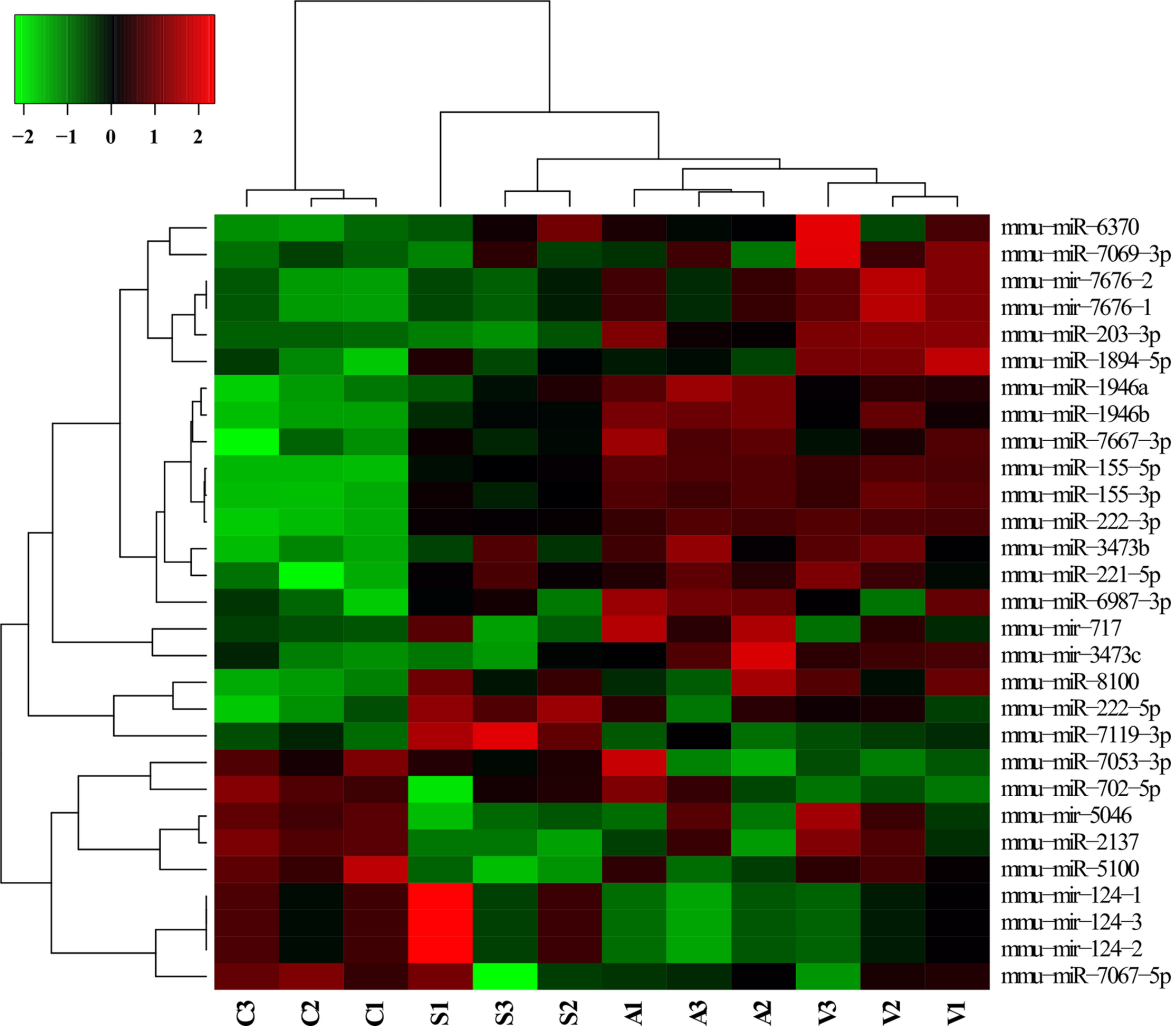
Heatmap of miRNAs modulated by macrophages at 8h of infection by different strains of *Leptospira* spp. Heatmap shows the average signal of 29 miRNAs/treatment (n = 3/treatment; p-value <0.01; linear fold change ±1.5).

**Fig 4:**
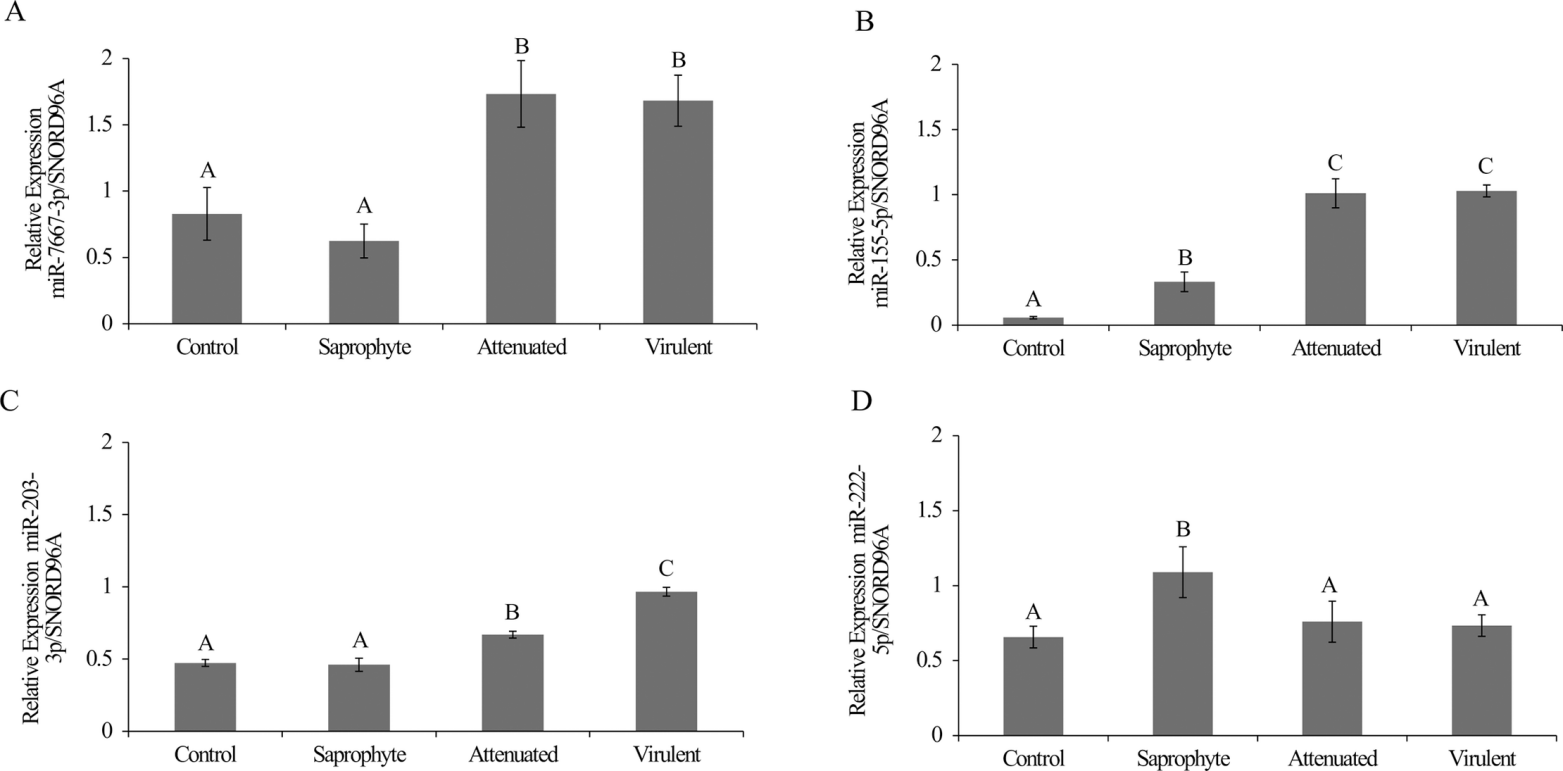
qRT-PCR of miRNA expression levels in macrophages infected with different strains of *Leptospira* compared to non-infected controls. **A)** Relative expression of miR-7667-3p, **B)** Relative expression of miR-155-5p, **C)** Relative expression of miR-203-3p, **D)** Relative expression of miR-222-5p. Different superscript letters differ significantly (p<0.05).

**Fig 5:**
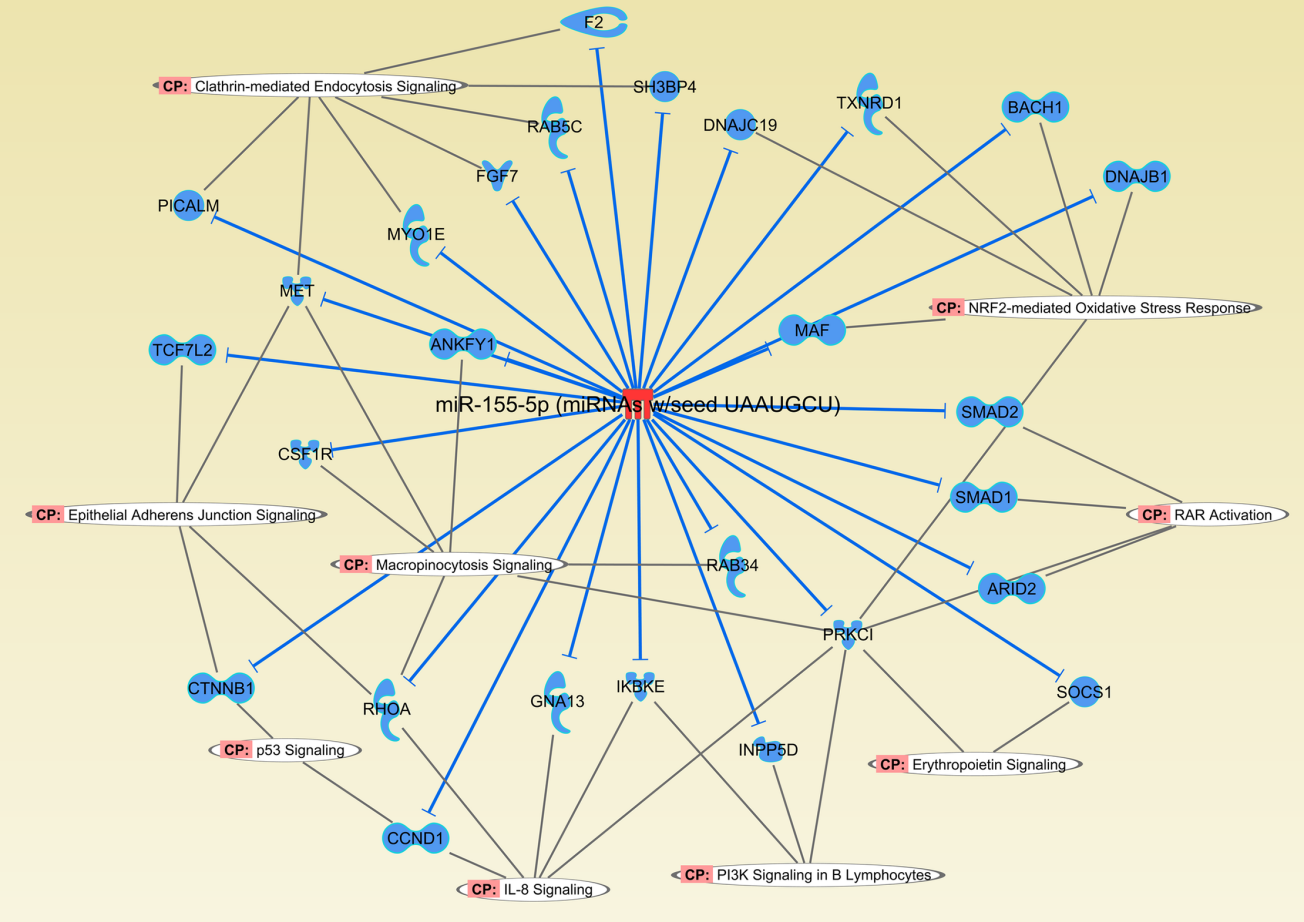
Canonical pathways of miR-155-5p targets identified in virulent treatment compared to non-infected control. Network shows potential downregulated targets of mmu-miR-155-5p participating in biological pathways identified only in virulent treatment when compared to control. In red, miR-155 was upregulated and blue indicates the potentially downregulated targets of miR-155-5p in macrophages infected with virulent *L*. *interrogans* following 8h of infection *in vitro*. (CP = Canonical pathways).

In total, we identified 29 miRNAs that were modulated in macrophages after 8h of infection with different strains of *Leptospira* spp (fold change ± 1.5; p <0.01). When compared to non-infected control cells, 17 miRNAs were significantly altered (15 upregulated and 2 downregulated) following infection with the virulent strain, 16 miRNAs were modulated (12 upregulated and 4 downregulated) as a response to the attenuated strain, and 9 miRNAs were altered by infection with the saprophyte strain (5 upregulated and 4 downregulated) (Fig 1). The intersection of treatments in the Venn diagram shows that three miRNAs are modulated by all strains. MiRNAs were also modulated in a strain-specific manner, where 7 miRNAs were modulated specifically by the virulent strain, 7 by the attenuated and 5 only by the saprophyte bacteria (Fig 2). Average signals (log2) of samples were hierarchically clustered using Pearson´s correlation and complete-linkage, we observed a clustering of samples based on species and virulence, with the virulent and attenuated strains clustering closer together, followed by the saprophyte strain (Fig 3), and all infected samples clearly separating from non-infected controls. Validation of chosen regulated miRNAs by quantitative realtime PCR (miR-155-5p; miR-7667-3p; miR-203-3p and 222-5p), corroborated the microarray results with respective correlation coefficients between techniques of 0.99, 0.79, 0.92 and 0.99 (Fig 4). The highest fold change was observed for miR-155-5p with an upregulation of >12 fold for *L*. *interrogans* and >5 fold for the saprophyte *L*. *biflexa*, when compared to non-infected cells. In Fig 5, we depict the significant canonical pathways for miR-155-5p targets, identified in the virulent treatment compared to noninfected control cells.

### miRNA target prediction and functional enrichment

For prediction of target genes to the differentially expressed miRNAs in all treatments, we used the tool miRNA Target Filter present in the IPA software. We utilized databases from miRecords, Tarbase, TargetScan, and the Ingenuity Knowledge Base, and filtered for targets with high prediction and that have been experimentally observed only. In Table 1, we demonstrate that 10 out of the 29 differentially expressed miRNAs have predicted mRNA targets. To identify the relationships, mechanisms, functions, and pathways relevant to the list of target genes, we employed the feature Core Analysis available in the IPA software package with values of significance–log (BH corrected p-value)>1.3. From our list of predicted genes for each treatment, we report 21 and 5 canonical pathways for virulent and attenuated treatment, respectively (Tables 2 and 3). For the saprophyte treatment, specific miRNAs did not have predicted targets. Further, only the virulent treatment had specific pathways identified.

**Table 1:** Number of miRNA targets to the differentially expressed miRNAs following infection with virulent, attenuated or saprophyte *Leptospira*. **(**CT **=** control; FC = fold change).

miRNAs			Virulent x CT				Attenuated X CT				Saprophyte X CT			Number of Targets	
			FC	p-value			FC	p-value			FC	p-value			
miR-155-3p			**14.3**	**0.000066**			**14.4**	**0.000006**			**6.3**	**0.000286**		2	
miR-155-5p			**12.1**	**0.000004**			**12.4**	**2.46E-07**			**5.9**	**0.000007**		224	
miR-221-5p			**1.8**	**0.012387**			**1.7**	**0.005452**			**1.6**	**0.009546**		298	
miR-1946b			**1.5**	**0.002698**			**2.0**	**0.000014**			1.4	0.000770		-	
miR-3473b			**1.6**	**0.003419**			**1.5**	**0.006212**			1.2	0.038841		-	
miR-1946a			**1.5**	**0.00278**			**1.8**	**0.001035**			1.1	0.490372		-	
miR-203-3p			**2.3**	**0.000001**			**1.5**	**0.019451**			-1.1	0.312029		5	
miR-222-3p			**1.5**	**0.000009**			**1.5**	**0.00003**			1.3	0.000024		177	
miR-7667-3p			**3.2**	**0.021577**			**4.7**	**0.005985**			2.4*	0.031972		219	
miR-7069-3p			**1.6**	**0.010383**			1	0.350463			1	0.488653		189	
mir-3473c			**1.5**	**0.009386**			1.65	0.051234			1	0.868143		-	
mir-7676-1			**1.5**	**0.001562**			1.3	0.018375			1.1	0.092322		-	
mir-7676-2			**1.5**	**0.001562**			1.3	0.018375			1.1	0.092322		-	
miR-1894-5p			**1.5**	**0.00436**			1	0.096391			1.2	0.082984		-	
miR-702-5p			**-1.5**	**0.001429**			-1	0.416406			-1.1	0.162260		64	
miR-7053-3p			**-1.5**	**0.006674**			-1.7	0.411229			-1.1	0.162706		-	
miR-6987-3p			1.3	0.204396			**1.8**	**0.006432**			1.3	0.242025		78	
miR-6370			2	0.076150			**1.6**	**0.0016**			1.71	0.068848		-	
mir-717			1	0.441789			**1.5**	**0.010607**			-1	0.799834		-	
miR-7067-5p			-1.3	0.127036			**-1.8**	**0.010926**			-1.9	0.218096		730	
mir-124-3			-1.4	0.107360			**-1.9**	**0.010232**			1	0.697852		-	
mir-124-1			1	0.580372			**-1.9**	**0.010232**			1	0.697852		-	
mir-124-2			-1.4	0.107370			**-1.9**	**0.010232**			-1	0.909340		-	
miR-222-5p			1.5*	0.041164			1.5	0.111593			**1.6**	**0.004998**		-	
miR-7119-3p			1	0.589778			- 1	0.867308			**-1.5**	**0.005767**		-	
miR-5100			-1	0.183061			1.2	0.067205			**-1.5**	**0.000297**		-	
miR-2137			- 1	0.453161			-1.3	0.073805			**-1.6**	**0.0031**		-	
mir-5046		- 1	0.666151			-1.6	0.122672			**1.6**	**0.007021**			-	
miR-8100		**1.7**	**0.005375**			1.25	0.110823			**1.58**	**0.007021**			-	

**Table 2:** Common canonical pathways identified using the targets of miRNAs expressed by macrophages infected with different strains of *Leptospira* spp. Core analysis from IPA (Ingenuity Pathway Analysis) software was used to identify significant pathways trough B-H Multiple testing correction -log(B-H p-value)>1.3.

Ingenuity Canonical Pathways	Contrast	-log(B-H p-value)	Genes in Pathway
Molecular Mechanisms of Cancer	Virulent x Control	3.13	41/376
	Attenuated x Control	1.96	48/376
Fcγ Receptor-mediated Phagocytosis in Macrophages and Monocytes	Virulent x Control	1.98	15/93
	Attenuated x Control	2.01	19/93
PI3K/AKT Signaling	Virulent x Control	1.91	17/125
	Attenuated x Control	1.96	22/125
PTEN Signaling	Virulent x Control	1.80	16/119
	Attenuated x Control	2.01	22/119
Role of Macrophages, Fibroblasts and Endothelial Cells in Rheumatoid Arthritis	Virulent x Control	1.56	29/311
	Attenuated x Control	1.55	40/311

**Table 3:** Specific canonical pathways identified using the targets of miRNAs modulated by macrophages infected with virulent strain (*L*. *interrogans* serovar Copenhageni L1-130). Core analysis from IPA (Ingenuity Pathway Analysis) software was used to identify significant pathways trough B-H Multiple testing correction log(B-H p-value) >1.3.

Ingenuity Canonical Pathways	-log(B-H p-value)	Genes in Pathway
PI3K Signaling in B Lymphocytes	1.98	18/130
NRF2-mediated Oxidative Stress Response	1.62	21/193
IL-8 Signaling	1.91	23/197
Clathrin-mediated Endocytosis Signaling	1.56	21/199
p53 Signaling	1.70	15/111
Ovarian Cancer Signaling	1.36	16/144
Osteoarthritis Pathway	1.36	21/210
Small Cell Lung Cancer Signaling	1.56	12/85
Tec Kinase Signaling	1.36	18/170
Erythropoietin Signaling	1.62	12/81
Macropinocytosis Signaling	1.62	12/81
Epithelial Adherens Junction Signaling	1.56	17/146
Gα12/13 Signaling	1.36	15/131
Regulation of IL-2 in Activated and Anergic T Lymphocytes	1.36	29526
Pancreatic Adenocarcinoma Signaling	1.36	14/118
RAR Activation	1.88	22/190

Five biological processes, Molecular Mechanisms of Cancer, Fcγ Receptormediated Phagocytosis in Macrophages, PI3K/AKT Signaling, PTEN Signaling and Role of Macrophages in Rheumatoid Arthritis are potentially regulated by miRNAs in response to *L*. *interrogans* infection, regardless of virulence. These five pathways were therefore classified as common to *L*. *interrogans*. In regards to specific processes, 16 pathways were identified only in response to the virulent strain compared to noninfected controls.

## Discussion

Despite its prevalence, morbidity and mortality rates, pathogenicity of leptospirosis is still largely unknown. To better understand the processes involved in the host-pathogen interplay of *Leptospira* infection, we carried out, for the first time, a global evaluation of microRNA modulation of murine macrophages infected with different strains of *Leptospira* spp at 8h post-infection *in vitro*. Here, we compared miRNA profiles to address whether *Leptospira* affect macrophageal miRNA expression, if this modulation is species specific and if bacterial virulence plays a role in this modulation.

In Fig 3, we show that *Leptospira* spp, regardless of species or virulence, modulate the miRNAs mmu-miR-155-5p, mmu-miR-155-3p and mmu-miR-221-5p. Notwithstanding, specific miRNAs signatures were also obtained for each strain, and they differed within species based on virulence. Differential host response has been previously suggested to be associated to protein differences between the strains. In fact, Haake et al., [34] reported that attenuated *L*. *interrogans* cultures expressed different proteins and LPS profiles when compared to virulent cultures. In addition, Picardeau et al. [35], compared the genomic sequence of saprophyte *L*. *biflexa* with that of the pathogenic strain, *L*. *interrogans*, identifying differences such as a larger number of genes encoding proteins containing leucine-rich repeat (LRR) domains in the pathogenic strain, shown to be involved in attachment and invasion of host cells in other bacteria. Therefore, it is not surprising that differing bacterial genomic and protein profiles elicit different responses in their host cells. Further, Xu et al. [36] demonstrated significant differences in gene expression, particularly in genes related to antigen processing and presentation, regulation of membrane potential, cell migration, cytoskeleton organization and biogenesis are mostly up-regulated in murine macrophage cells, following infection by different species of *Leptospira*. Our current results indicate that, beyond the previously reported genomic and transcriptional differences, control of gene expression by means of post-transcriptional modifications may be dependent on species and virulence in leptospiral infection.

Among the miRNAs identified in our study as commonly regulated by the genus *Leptospira* sp, independent of virulence, is mmu-miR-155-5p. This miRNA is known to be upregulated in inflammatory processes such as rheumatoid arthritis, cancer, cardiovascular disease, as well as in other bacterial infections in macrophages like *Listeria*, *Salmonella*, *Helicobacter* and *Mycobacteria* [37] reviewed by [31,38]. MiR155 has a considerable number of mRNA targets, and all canonical pathways identified here, in virulent and attenuated treatments, have highly predicted and experimentally observed targets of mmu-miR-155 (S1 and S2 Tables), suggesting an important role for miR-155 as a post-transcriptional regulator in leptospiral induced host response.

Macrophages are fundamental against leptospiral infection. They have different receptors that activate a plethora of responses, such as phagocytosis, cytokine/chemokine production and antigen presentation [39–41]. The cross-linking of IgGs (immunoglobulin-g) with Fc receptors in macrophages initiates crucial cellular events for host immune response. The pathway Fc-gamma receptors in macrophages, play an important role in recognizing IgG-coated pathogen targets during the phagocytosis process in the host [42]. Our target analysis identified previously reported genes in this pathway, significantly identified following virulent and attenuated infection, such as SHIP-1, VAV3 and VAMP3 suggested to be downregulated by mmumiR-155-5p, and PTEN, downregulated by mmu-miR-222-3p, involved in phagosome formation and recycling of cell membrane, respectively. Phagocytosis is vital for internalization of leptospires, and previous work has indicated that one of the resistance mechanism of pathogenic *Leptospira* in the host, is evasion of the alternative and classical complement system pathways [43]. VAV3, is a potential target to miR-155-5p, and downregulation of this gene can cause inhibition in B- and T-cell development and activation, given its involvement in activating pathways that lead to actin cytoskeletal rearrangements and greater cellular movement [44]. More studies are needed to correlate *Leptospira* evasion and VAV3 function. Inositol polyphosphate-5-phosphatase D (INPPD5 or SHIP-1) is a well established target for mmu-miR-155-5p [37] and this correlation is associated with cell proliferation. Increased expression of mmu-miR-1555p, with a resulting decrease of INPPD5, promotes transcription of major proinflammatory cytokines in macrophages [45–46]. Restoration of INPPD5 levels is related to an inhibition in PI3K-AKT signaling and anti-inflammatory response in raw264.7 cells and primary bone marrow-derived macrophages (BMDMs), as reported in bowel disease [46]. It is tempting to hypothesize that, at early infection (8h in this study), INPPD5 is downregulated by mmu-miR-155-5p, leading to the production of major pro-inflammatory cytokines, such as 1L-1α and TNF-α, previously observed in macrophages [36], as a first response to infection by *Leptospira*.

Another common pathway identified in this study, between attenuated and virulent strains, was PI3K/AKT signaling with several target genes for mmu-miR-1555p involved in pro-inflammatory response. This pathway is responsible for B cell development trough activation of several genes. Inhibited PI3K signaling leads to immunodeficiency, autoimmunity activation and leukemia [47–48]. Cheung et al., [49] have shown that IL-10 inhibits miR-155, but this process is dependent on the presence of INPPD5 (a target to miR-155), and also that the activation of PI3K/AKT signaling abolished IL-10-inhibition of miR-155, resulting in an increase of miR-155 [49]. It is plausible to infer that miR-155 can be dependent of PI3K/AKT signaling to promote inflammation in *L*. *interrogans* infection, and that mmu-miR-155 could be a master regulator of pro-inflammatory process in leptospiral infection and a potential therapy target or biomarker.

Another goal in the present study was to identify differences in macrophageal response to strains, varying only in virulence. We have identified 16 specific canonical pathways potentially regulated by miRNAs modulated following infection with virulent *L*. *interrogans*. Cellular movement and cell-to-cell signaling interactions are functions related with the canonical pathways IL-8 (CXCL8) signaling, Tec kinase signaling, Epithelial adherens junction signaling and Gα12/13 signaling. It is well know that *Leptospira* causes changes in adherens junctions and endothelial cells increasing vascular permeability, potentially leading to severe illness [50]. The small GTPase RhoA (ras homolog family member A), a protein that regulates actin cytoskeleton and the remodeling of cell junctions, appears in most of the pathways mentioned above, as a direct target of miR-155-5p. In a recent study, Sato & Coburn [50] found a slight elevation of RhoA protein levels in endothelial cells following infection of both virulent and saprophyte *Leptospira* strains. In our macrophage cells, we observed an increase of miR-155-5p, which could lead to a decrease in RhoA, contrary to what was observed by Sato & Coburn in the endothelial cell line. This could be due to a cell type difference, to a difference in time of infection (8h in our study versus 24h in the aforementioned study), or to a difference in regulation of RhoA between the cell types.

A very common occurrence in patients with leptospirosis is a coagulation disorder causing lung hemorrhage [4]. The mechanism behind this *Leptospira*-induced hemorrhage is not fully understood. Fernandes *et al*, [51] provide evidence that lower serum levels of prothrombin and antithrombin III in patients with the disease is related to the observed hemorrhage. Liver-produced prothrombin remains inactive in circulation until being proteolytically cleaved to form thrombin to start clot formation by converting fibrinogen to fibrin. Here, we can suggest that Prothrombin (Coagulation Factor II) is potentially downregulated by miR-155-5p, suggesting a role for epigenetically mediated post-transcriptional control of clot formation in leptospirosis patients. In fact, recent studies support the idea that miR-155-5p can be secreted to act as modulators elsewhere. Wang *et al*. [52] have just shown that macrophages secrete miR-155-5p that can act as paracrine regulators of inflammation during cardiac injury. Alexander *et al*. [53] have also demonstrated that miR-155 present in exosomes can pass between immune cells *in vivo* and promote endotoxin-induced inflammation in mice.

Pathogen invasion into host cells is crucial for pathogenicity. Through phagocytosis, macrophages can kill the invading bacteria early in the process of infection. Both macropinocytosis signaling and clathrin-mediated endocytosis signaling are pathways significantly identified following infection with the virulent strain. In the macropinocytosis process, two genes appear to be down-regulated, Colony Stimulating Factor 1 Receptor (CSF1R), a target of miR-155-5p and SRC (Proto-oOncogene, nNonReceptor Tyrosine Kinase (SRC), targeted by mmu-miR-203-3p. CSF1R is a type III tyrosine kinase receptor, involved in cell proliferation and survival, and when phosphorylated, this receptor activates SRC kinases to initiate the signal cascade required for macropinocytosis process [54–55]. Therefore these miRNAs have potential to control initial signaling cascades of macropinocitosis.

During phagocytosis, one of the most effective weapons used by macrophages to kill invading leptospires is nitric oxide (NO) and reactive oxygen species (ROS), which induce an antimicrobial response [56–57]. On the other hand, an excessive production of O_2_ by macrophages can affect homeostasis [56]. This burden of intracellular oxygen demand by macrophages to kill pathogens has important collateral effects that can contribute to the inflammatory process through hypoxia in tissues [58] and DNA damage [59].

Further, Luo *et al*., [56] reported that Erythropoietin signaling, significantly identified in our study as a response to virulent infection, has a vital function in regulation of acute inflammatory conditions in hypoxia. This process of inflammation regulation appears to be inhibited in our acute infection, which could ultimately be associated with the exacerbated inflammation commonly seen in leptospirosis.

Hu and colleagues [59] report that leptospiral infection in macrophages induces cell cycle arrest dependent of p53/p21. Here we identified that the target pathway p53 signaling is regulated, following virulent infection, by modulation of miRNAs. This pathway can be activated by DNA damage, hypoxia, cytokines, metabolic changes, viral infection or oncogenes [60–61], and our study adds bacterial infection by *Leptospira* sp as another activator. This pathway triggers three important processes in the host cell, cell cycle arrest for DNA repair, apoptosis and cell survival. Here we found that BCL2 (anti-apoptotic gene) is potentially downregulated by mmu-miR-7667-3p following infection with *L*. *interrogans*, leading us to suggest that cell survival could be at risk following *L*. *interrogans* infection of macrophages.

Lastly, another virulent specific pathway identified in our study, the retinoic acid receptor (RAR) activation, has not been previously described in leptospirosis. This canonical pathway is related to development, differentiation, apoptosis and homeostasis, mostly participating in phosphorylation of several signaling pathways, as reviewed by [62], and also has fundamental importance in acquired and adaptative immune responses, with an important role in clonal expansion, differentiation, survival of pathogen-specific CD8 T cells, and bacterial clearance, as confirmed by a knockout model of RAR in mice [63]. These nuclear receptors act on recruitment of the transcriptional machinery to DNA response elements, regulating other complexes like nuclear factor kappa B (NF-κB complex) [62], SMAD complex [63], and also interact with other signaling pathways like PI3K/Akt and PTEN [62], which are vital to immune response. Here we found genes like Mothers Against Decapentaplegic Homolog (SMAD1/2) as a predicted target to mmu-miR-155-5p, SMAD7/9 targeted by miR- 7667-3p, TGF-β, that indirectly increases activation of SMAD complexes and is a target of mmu-miR-7069-3p and, finally, nuclear factor kappa B (NF-κB complex), a predicted target of mmu-miR-155-5p [64]. We suggest that upregulation of these miRNAs following macrophageal infection by *L*. *interrogans* can negatively regulate immune response to virulent leptospires through modulation of RAR activation. Interestingly, it has been demonstrated that vitamin A, a ligand of RAR, has antimicrobial activity against monocytes infected with *Mycobacterium tuberculosis*, in a mechanism dependent of intracellular cholesterol transporter 2 (NPC2) [65], raising the question as to whether this effect extends to other bacterial infections.

Our data provides the first evidence that host miRNAs are regulated by *Leptospira* infection in macrophages in a virulence- and species-specific manner *in vitro*. A large number of the identified miRNA targets participate in key processes involved in the immune response. Characterization of this regulatory network may help to understand the pathogenesis of leptospirosis and to identify miRNAs as biomarkers of infection or as targets for therapy. In conclusion, we suggest that post-transcriptional regulation by miRNAs play a role in the host’s response to leptospirosis infection.

## Methods

### Bacterial strains

Three types of bacterial samples were utilized in this study, *Leptospira interrogans* serovar Copenhageni strain FIOCRUZ-L1-130, as a virulent strain; the pathogenic culture-attenuated *L*. *interrogans* serovar Copenhageni strain M-20; and *Leptospira biflexa* serovar Patoc strain FIOCRUZ-Patoc I as a saprophyte strain.

Bacteria were maintained in Fletcher semi solid culture medium, and incubated at 30°C. To restore bacterial virulence in strain L1-130, 1mL of cultured bacteria was inoculated intraperitoneally in hamsters (*Mesocricetus auratus*) and later recovered from kidneys. Attenuated strain did not undergo intraperitoneal inoculation in hamsters [66]. The inoculum was quantified using the camera of Petroff-Hausser.

### Ethics statement

Bacterial samples were provided by Laboratory of Preventive Veterinary Medicine of University of São Paulo (USP). Production of these samples were in accordance with Ethics Committee for Animal Use (FOA-FMVA UNESP), under protocol number 2015–00895. Following bacterial arrival, no animal experimentation was performed in the experiments described herein.

### Macrophage culture

Murine monocyte-macrophage cells (*Mus musculus* monocyte-macrophage cell line J774A.1), provided by the Paul Ehrlich cell bank, Rio de Janeiro, Brazil, was maintained in RPMI1640 media (Sigma, USA) supplemented with 10% heat-inactivated fetal bovine serum (Gibco, USA), 100ug/mL streptomycin (Sigma Chemical Co St. Louis, MO), 0.03% Lglutamine solution (Sigma) and 100 UI/mL of penicillin. Cells were incubated at 37° C, 5% CO2 until formation of a confluent monolayer in 6-well cell culture plates (3cm/well).

### Infection of macrophages

Cultured cells were washed three times with sterile phosphate buffer solution (pH 7,2) for removal of antibiotics and non-adherent cells. *L*. *interrogans* and *L*. *biflexa* were harvested by centrifugation and the pellet was resuspended in RPMI-1640 media (Sigma), and 100:1 bacteria:cell were added to macrophages at confluency (MOI of 100), as previously described [24]. Treatments, performed in three biological replicates, were carried as follows: infection of macrophages with a virulent strain (*L*. *interrogans*), infection with attenuated strain (*L*. *interrogans*), saprophyte strain (*L*. *biflexa*) and non-infected macrophages (control). All treatments were incubated in fresh RPMI medium, without antibiotics, for 8h at 37° C, 5% C0_2_. Rate of infection did not differ between strains (78, 85 e 80% for saprophyte, attenuated and virulent, respectively). Following this period, RNA extraction was immediately performed as described below.

### RNA extraction and quantification

Total RNA was extracted from macrophages with a miRVana miRNA Isolation Kit (Ambion, Austin, TX, USA) according to the manufacturer’s instructions. RNA samples were immediately stored at -80°C. Quantification was performed using NanoDrop (ND-2000 spectrophotometer, Thermo Scientific, Wilmington, DE, USA) and quality of samples was assessed using capillary electrophoresis (Bioanalyzer 2100 Agilent, Santa Clara, CA, USA). All samples used for microarray analysis had a RIN of 10.

### Microtranscriptome array

MicroRNA profiles were obtained from 250ng/sample of total RNA (RIN 10) using the FlashTag Biotin HSR RNA Labeling Kit, and the Affymetrix miRNA 4.1 Array strip (Affymetrix, Santa Clara, California, EUA), containing 3195 murine specific probes of miRNA, according to the manufacturer’s instructions. A recommended ELOSA quality control assay was run for all samples, and hybridization of samples to the strips was carried at 48°C for 20h. Following this period, strips were processed and scanned using the GeneAtlas System (Affymetrix). Raw intensity values were background corrected, log2 transformed and then quantile normalized by the software Expression Console (Affymetrix) using the Robust Multi-array Average (RMA) algorithm. Data files were deposited at Gene Expression Omnibus (GSE105104). Statistical analysis was performed in the TAC software (Affymetrix) by ANOVA (fold change ± 1.5, p <0.01).

### Identification of microRNA targets and functional enrichment

For identification of target genes we employed the miRNA Target Filter Analysis from the Ingenuity Pathway Analysis (IPA) software (Qiagen). For selection, we opted to use conservative filters, allowing only experimentally observed and highly predicted targets to be selected (Supplementary table). For the identification of canonical pathways potentially regulated by the differentially expressed miRNAs, we employed the Benjamini-Hochberg (BH) correction for multiple testing (BH corrected p<0.05).

### Validation of microarray results by qRT-PCR

For validation of miRNA expression in infected macrophages (saprophyte, attenuated and virulent strains) and non-infected control macrophages, we employed the miScript miRNA PCR System (Qiagen-Valencia, CA, USA) for preparation of cDNA and realtime PCR, according to manufacturer´s instructions. Validated inventoried primers employed were purchased from Qiagen. PCR was performed using a Stratagene QPCR System Mx3005P (Agilent Technologies, Santa Clara, CA, USA), following instructions on the miScript miRNA PCR System´s manual. Expression levels were determined using standard curves for all miRNAs at each individual run, and the expression of candidate miRNAs is presented as a ratio to the control miRNA SNORD96A.

### Statistical analysis

Differential expression of each miRNA was determined by ANOVA with two criteria, a fold change of ±1.5 comparing all infected groups to the non-infected control and p-value<0.01. Real time PCR data was analyzed using least-squares analysis of variance and the general linear model procedures of SAS (SAS Institute, Cary, NC, USA; p<0.01). Comparison of means was done using Duncan’s multiple range test, and significance was set at p<0.05.

### Accession numbers

mmu-miR-155-3p (MIMAT0016993); mmu-miR-155-5p (MIMAT0000165); mmu-miR-221-5p (MIMAT0016070); mmu-miR-1946b (MIMAT0009443); mmu-miR-3473b (MIMAT0020367); mmu-miR-1946a (MIMAT0009412); mmu-miR-203-3p (MIMAT0000236); mmu-miR-222-3p (MIMAT0000670); mmu-miR-7667-3p (MIMAT0029841); mmu-miR-7069-3p (MIMAT0028045); mmu-mir-3473c (MI0018015); mmu-mir-7676-1 (MI0025017); mmu-mir-7676-2 (MI0025018); mmu-miR-1894-5p (MIMAT0007877); mmu-miR-702-5p (MIMAT0022931); mmu-miR-7053-3p (MIMAT0028011); mmu-miR-6987-3p (MIMAT0027877); mmu-miR-6370 (MIMAT0025114); mmu-mir-717 (MI0004704); mmu-miR-7067-5p (MIMAT0028038); mmu-mir-124-3 (MI0000150); mmu-mir-124-1 (MI0000716); mmu-mir-124-2 (MI0000717); mmu-miR-222-5p (MIMAT0017061); mmu-miR-7119-3p (MIMAT0028136); mmu-miR-5100 (MIMAT0020607); mmu-miR-2137 (MIMAT0011213); mmu-miR-5046 (MIMAT0020540); mmu-miR-8100 (MIMAT0031403).

## Supporting information

S1 Table Common pathways with respective miRNAs and targets obtained from IPA software. (DOCX)

S2 Table Specific pathways with respective miRNAs and targets obtained from IPA software. (DOCX)
