# Supplementary material for: Characterization of the microtranscriptome of macrophages infected with virulent, attenuated and saprophyte strains of Leptospira spp
Source: PLoS Negl Trop Dis. 2018 Jul 6;12(7):e0006621. doi: 10.1371/journal.pntd.0006621 (PMC6051669; doi:10.1371/journal.pntd.0006621)
Supplement: S1 Table — (DOCX) [file pntd.0006621.s001.docx]

**Supplementary table.**

**S1. Common pathways with respective miRNAs and targets obtained from IPA software.**

| **Pathway** | **ID** | **Confidence** | **Symbol** |
| --- | --- | --- | --- |
| Fc-gammaR-mediated phagocytosis in macrophages | mmu-miR-155-5p | High (predicted) | ACTA1 |
|  | mmu-miR-155-5p | Experimentally Observed | SHIP-1 |
|  | mmu-miR-155-5p | Experimentally Observed,High (predicted) | PRKCI |
|  | mmu-miR-155-5p | High (predicted) | RPS6KB1 |
|  | mmu-miR-155-5p | Experimentally Observed | VAMP3 |
|  | mmu-miR-155-5p | High (predicted) | VAV3 |
|  | mmu-miR-7069-3p | High (predicted) | GAB2 |
|  | mmu-miR-203-3p | Experimentally Observed | SRC |
|  | mmu-miR-222-3p | High (predicted) | ACTR3 |
|  | mmu-miR-222-3p | Experimentally Observed,High (predicted) | PIK3R1 |
|  | mmu-miR-222-3p | Experimentally Observed | PTEN |
|  | mmu-miR-221-5p | High (predicted) | PRKCZ |
|  | mmu-miR-7667-3p | High (predicted) | CBL |
|  | mmu-miR-7667-3p | High (predicted) | FYB |
|  | mmu-miR-7667-3p | High (predicted) | GAB2 |
|  | mmu-miR-7667-3p | High (predicted) | RAC2 |
|  | mmu-miR-7067-5p | High (predicted) | PLA2G6 |
|  | mmu-miR-7067-5p | High (predicted) | PXN |
|  | mmu-miR-7067-5p | High (predicted) | VASP |
|  | mmu-miR-6987-3p | High (predicted) | DGKB |
| PI3K/AKT Signaling | mmu-miR-155-5p | Experimentally Observed | CCND1, CTNNB1, INPP5D |
|  | mmu-miR-155-5p | Experimentally Observed,Moderate (predicted) | IKBKE, RHEB |
|  | mmu-miR-7069-3p | Experimentally Observed | KRAS |
|  | mmu-miR-222-3p | Experimentally Observed | FOXO3, PTEN |
|  | mmu-miR-222-3p | Experimentally Observed,High (predicted) | CDKN1B, PPP2R2A, PIK3R1 |
|  | mmu-miR-155-5p | High (predicted) | RPS6KB1 |
|  | mmu-miR-7069-3p | High (predicted) | GAB2 |
|  | mmu-miR-221-5p | High (predicted) | CCND1, PRKCZ |
|  | mmu-miR-7667-3p | High (predicted) | BCL2, GRB2, EIF4E, GAB2 |
|  | mmu-miR-7067-5p | High (predicted) | EIF4EBP1, SFN, PPP2R5D, PPP2R5B, ITGA3, FOXO3 |
|  | mmu-miR-6987-3p | High (predicted) | YWHAH |
| Molecular mechanism of Cancer | mmu-miR-155-5p | Experimentally Observed | CCND1, FADD, CTNNB1, GNA13, PMAIP1, RHOA, SMAD2 |
|  | mmu-miR-155-5p | Experimentally Observed,Moderate (predicted) | SMAD1, TAB2 |
|  | mmu-miR-155-5p | Experimentally Observed,High (predicted) | PRKCI |
|  | mmu-miR-155-5p | High (predicted) | APAF1, FOS, TCF4 |
|  | mmu-miR-7069-3p | Experimentally Observed | KRAS |
|  | mmu-miR-7069-3p | High (predicted) | BCL2L11, WNT8B, CASP7, CDKN2D, GAB2, RND2, TGFB3 |
|  | mmu-miR-203-3p | Experimentally Observed | ABL1, SRC |
|  | mmu-miR-222-3p | Experimentally Observed | BCL2L11 |
|  | mmu-miR-222-3p | Experimentally Observed,Moderate (predicted) | APAF1, BBC3 |
|  | mmu-miR-222-3p | Experimentally Observed,High (predicted) | DIRAS3, CDKN1B, FOS, PIK3R1 |
|  | mmu-miR-222-3p | High (predicted) | CDKN2B |
|  | mmu-miR-7667-3p | High (predicted) | ARHGEF7, SMAD9, SMAD7, RAC2, BCL2, CBL, GAB2, GRB2 |
|  | mmu-miR-7067-5p | High (predicted) | BAK1, WNT5B, TLR9, TGFBR2, SMAD7, PRKAR1B, PAK4, ITGA3, GNAI2, FZD9, FADD, CAMK2G, BCL2L11, WNT7B |
|  | mmu-miR-221-5p | High (predicted) | CAMK2A, SUV39H1, CCND1, FADD, GNA14, NOTCH1, PRKCZ, RHOD |
|  | mmu-miR-6987-3p | High (predicted) | HIF1A. WNT7A |
| PTEN Signaling | mmu-miR-155-5p | Experimentally Observed | CCND1, INPP5D |
|  | mmu-miR-155-5p | Experimentally Observed,Moderate (predicted) | IKBKE |
|  | mmu-miR-155-5p | High (predicted) | RPS6KB1 |
|  | mmu-miR-7069-3p | High (predicted) | BCL2L11 |
|  | mmu-miR-7069-3p | Experimentally Observed | KRAS |
|  | mmu-miR-222-3p | Experimentally Observed | BCL2L11, PTEN, FOXO3 |
|  | mmu-miR-222-3p | Experimentally Observed,High (predicted) | CDKN1B, PIK3R1 |
|  | mmu-miR-222-3p | High (predicted) | KDR |
|  | mmu-miR-221-5p | High (predicted) | CCND1, PRKCZ |
|  | mmu-miR-7667-3p | High (predicted) | BCL2, RAC2, CBL, GRB2 |
|  | mmu-miR-7067-5p | High (predicted) | BCL2L11, DDR1, FOXO3, FOXO4, ITGA3, NGFR, TGFBR2, TNFRSF11A |
|  | mmu-miR-6987-3p | High (predicted) | YWHAH |
| Role of Macrophages, Fibroblasts and Endothelial Cells in Rheumatoid Arthritis | mmu-miR-155-5p | Experimentally Observed | CCND1, RHOA, RIPK1, CTNNB1, MYD88 |
|  | mmu-miR-155-5p | Experimentally Observed,High (predicted) | CEBPB, PRKCI |
|  | mmu-miR-155-5p | Experimentally Observed,Moderate (predicted) | IKBKE, SOCS1, TCF7L2 |
|  | mmu-miR-155-5p | High (predicted) | CSNK1A1, TCF4, FOS, IL36G |
|  | mmu-miR-7069-3p | Experimentally Observed | KRAS |
|  | mmu-miR-7069-3p | High (predicted) | PDGFB, WNT8B, PROK1 |
|  | mmu-miR-203-3p | Experimentally Observed | SOCS3, SRC |
|  | mmu-miR-222-3p | Experimentally Observed | ICAM1 |
|  | mmu-miR-222-3p | Experimentally Observed,High (predicted) | FOS, PIK3R1 |
|  | mmu-miR-222-3p | Experimentally Observed,Moderate (predicted) | MMP1 |
|  | mmu-miR-222-3p | High (predicted) | PLCL2, SOCS3, PPP3R1 |
|  | mmu-miR-221-5p | High (predicted) | CAMK2A, PRKCZ, CCND1, IRAK4 |
|  | mmu-miR-7667-3p | High (predicted) | GRB2, PROK1, BCL2, CBL, GRB2, SMAD9 |
|  | mmu-miR-7067-5p | High (predicted) | CALM1 (includes others), FZD9, IL1RN, ITGA3, MAP2K7, NGFR, TLR9, TNFRSF11A, WNT5B, WNT7B |
|  | mmu-miR-6987-3p | High (predicted) | CALM1 (includes others), WNT7A |
