# Supplementary material for: Characterization of the microtranscriptome of macrophages infected with virulent, attenuated and saprophyte strains of Leptospira spp
Source: PLoS Negl Trop Dis. 2018 Jul 6;12(7):e0006621. doi: 10.1371/journal.pntd.0006621 (PMC6051669; doi:10.1371/journal.pntd.0006621)
Supplement: S2 Table — (DOCX) [file pntd.0006621.s002.docx]

**Supplementary table.**

**S2.Specific pathways with respective miRNAs and targets obtained from IPA software.**

| **Pathway** | **ID** | **Confidence** | **Symbol** |
| --- | --- | --- | --- |
| **PI3K Signaling in B Lymphocytes** | mmu-miR-155-5p | High (predicted) | FOS, VAV3 |
|  |  | Experimentally Observed,Moderate (predicted) | IKBKE |
|  |  | Experimentally Observed | INPP5D |
|  |  | Experimentally Observed,High (predicted) | PRKCI |
|  | mmu-miR-7069-3p | High (predicted) | IRS4 |
|  |  | Experimentally Observed | KRAS |
|  | mmu-miR-203-3p | Experimentally Observed | ABL1 |
|  | mmu-miR-222-3p | Experimentally Observed,High (predicted) | FOS, PIK3R1 |
|  |  | Experimentally Observed | FOXO3, PTEN |
|  |  | High (predicted) | PLCL2, PLEKHA2, PPP3R1 |
|  | mmu-miR-221-5p | High (predicted) | CAMK2A, PRKCZ, CD180 |
|  | mmu-miR-7667-3p | High (predicted) | CBL, CD19, CD180 |
| **IL-8 Signaling** | mmu-miR-155-5p | Experimentally Observed | CCND1, GNA13, RHOA |
|  |  | High (predicted) | FOS, RPS6KB1 |
|  |  | Experimentally Observed,Moderate (predicted) | IKBKE |
|  |  | Experimentally Observed,High (predicted) | PRKCI |
|  | mmu-miR-7069-3p | Experimentally Observed | KRAS |
|  |  | High (predicted) | PROK1, RND2 |
|  | mmu-miR-203-3p | Experimentally Observed | SRC |
|  | mmu-miR-222-3p | Experimentally Observed,High (predicted) | DIRAS3, FOS, PIK3R1 |
|  |  | Experimentally Observed | ICAM1 |
|  |  | High (predicted) | KDR |
|  | mmu-miR-221-5p | High (predicted) | CCND1, IRAK4, PRKCZ, RHOD |
|  | mmu-miR-7667-3p | High (predicted) | BCL2, GNG13, GRB2, MYL9, PROK1, RAC2 |
|  | mmu-miR-702-5p | High (predicted) | GNG13 |
| **RAR Activation** | mmu-miR-155-5p | Experimentally Observed,High (predicted) | ARID2, PRKCI |
|  |  | High (predicted) | FOS, RBP2 |
|  |  | Experimentally Observed,Moderate (predicted) | SMAD1 |
|  |  | Experimentally Observed | SMAD2 |
|  | mmu-miR-7069-3p | High (predicted) | CITED2, RXRB, SDR9C7, TGFB3 |
|  | mmu-miR-203-3p | Experimentally Observed | SRC |
|  | mmu-miR-222-3p | Experimentally Observed,High (predicted) | FOS, PIK3R1 |
|  |  | Experimentally Observed,Moderate (predicted) | MMP1 |
|  |  | Experimentally Observed | PTEN |
|  |  | High (predicted) | RBP2 |
|  | mmu-miR-221-5p | High (predicted) | ALDH1A2, IL3RA, NRIP2, PRKCZ |
|  | mmu-miR-7667-3p | High (predicted) | SMAD7, SMAD9, ZBTB16 |
|  | mmu-miR-702-5p | High (predicted) | GTF2H5 |
| **p53 Signaling** | mmu-miR-155-5p | High (predicted) | APAF1 |
|  |  | Experimentally Observed | CCND1, CTNNB1, PMAIP1 |
|  |  | Experimentally Observed,Moderate (predicted) | TNFRSF10A |
|  |  | Experimentally Observed,High (predicted) | TP53INP1 |
|  | mmu-miR-7069-3p | High (predicted) | CSNK1D, DRAM1 |
|  | mmu-miR-203-3p | Experimentally Observed | TP63 |
|  | mmu-miR-222-3p | Experimentally Observed,Moderate (predicted) | APAF1, BBC3 |
|  |  | Experimentally Observed,High (predicted) | PIK3R1 |
|  |  | Experimentally Observed | PTEN |
|  |  | High (predicted) | TP53BP2 |
|  | mmu-miR-221-5p | High (predicted) | CCND1, TP63 |
|  | mmu-miR-7667-3p | High (predicted) | BCL2, GRB2 |
| **NRF2-mediated Oxidative Stress Response** | mmu-miR-155-3p | Experimentally Observed | DNAJA2, DNAJB1 |
|  | mmu-miR-155-5p | High (predicted) | ACTA1, FOS |
|  |  | Experimentally Observed,High (predicted) | BACH1, PRKCI |
|  |  | Experimentally Observed | DNAJB1, MAF, DNAJC19, TXNRD1 |
|  | mmu-miR-7069-3p | Experimentally Observed | KRAS |
|  | mmu-miR-222-3p | Experimentally Observed,High (predicted) | FOS, PIK3R1 |
|  |  | Experimentally Observed,Moderate (predicted) | SOD2 |
|  | mmu-miR-221-5p | High (predicted) | DNAJB6, PRKCZ, GSTM4, GSTK1 |
|  | mmu-miR-7667-3p | High (predicted) | DNAJC5G, GPX2, GRB2, MAFF |
|  | mmu-miR-702-5p | High (predicted) | DNAJB5 |
| **Erythropoietin Signaling** | mmu-miR-155-5p | High (predicted) | FOS, RPS6KB1 |
|  |  | Experimentally Observed,High (predicted) | PRKCI |
|  |  | Experimentally Observed,Moderate (predicted) | SOCS1 |
|  | mmu-miR-7069-3p | Experimentally Observed | KRAS |
|  | mmu-miR-203-3p | Experimentally Observed | SOCS3, SRC |
|  | mmu-miR-222-3p | Experimentally Observed,High (predicted) | FOS, PIK3R1 |
|  | mmu-miR-222-3p | High (predicted) | SOCS3 |
|  | mmu-miR-221-5p | High (predicted) | EPO, PRKCZ |
|  | mmu-miR-7667-3p | High (predicted) | CBL, GRB2 |
| **Macropinocytosis Signaling** | mmu-miR-155-5p | Experimentally Observed | ANKFY1, MET, RHOA |
|  |  | Experimentally Observed,Moderate (predicted) | CSF1R, RAB34 |
|  |  | Experimentally Observed,High (predicted) | PRKCI |
|  | mmu-miR-7069-3p | Experimentally Observed | KRAS |
|  |  | High (predicted) | PDGFB |
|  | mmu-miR-203-3p | Experimentally Observed | SRC |
|  | mmu-miR-222-3p | Experimentally Observed,High (predicted) | PIK3R1 |
|  | mmu-miR-221-5p | High (predicted) | PRKCZ |
|  | mmu-miR-7667-3p | High (predicted) | GRB2 |
| **Clathrin-mediated Endocytosis Signaling** | mmu-miR-155-5p | High (predicted) | ACTA1 |
|  |  | Experimentally Observed | F2, SH3BP4, MYO1E, MET |
|  |  | Experimentally Observed,High (predicted) | FGF7, PICALM |
|  |  | Experimentally Observed,Moderate (predicted) | RAB5C |
|  | mmu-miR-7069-3p | High (predicted) | PDGFB, PROK1 |
|  | mmu-miR-203-3p | Experimentally Observed | SRC |
|  | mmu-miR-222-3p | High (predicted) | ACTR3, PPP3R1, HSPA8 |
|  | mmu-miR-222-3p | Experimentally Observed,High (predicted) | PIK3R1 |
|  | mmu-miR-221-5p | High (predicted) | APOL1, LDLR |
|  | mmu-miR-7667-3p | High (predicted) | APOL1, PROK1, PIP5K1C, CBL, GRB2 |
|  | mmu-miR-702-5p | High (predicted) | APOM |
| **Small Cell Lung Cancer Signaling** | mmu-miR-155-5p | High (predicted) | APAF1 |
|  |  | Experimentally Observed | CCND1 |
|  |  | Experimentally Observed,Moderate (predicted) | IKBKE |
|  | mmu-miR-7069-3p | High (predicted) | RXRB |
|  | mmu-miR-203-3p | Experimentally Observed | ABL1 |
|  | mmu-miR-222-3p | Experimentally Observed,Moderate (predicted) | APAF1, PTEN |
|  |  | Experimentally Observed,High (predicted) | CDKN1B, PIK3R1 |
|  |  | High (predicted) | CDKN2B |
|  | mmu-miR-221-5p | High (predicted) | CCND1, SUV39H1 |
|  | mmu-miR-7667-3p | High (predicted) | BCL2, GRB2 |
| **Epithelial Adherens Junction Signaling** | mmu-miR-155-5p | High (predicted) | ACTA1, TCF4 |
|  |  | Experimentally Observed | CTNNB1, RHOA, MET |
|  |  | Experimentally Observed,Moderate (predicted) | TCF7L2 |
|  | mmu-miR-7069-3p | Experimentally Observed | KRAS, NOTCH2 |
|  | mmu-miR-203-3p | Experimentally Observed | SRC |
|  | mmu-miR-222-3p | High (predicted) | ACTR3, NECTIN1, TUBA1A |
|  |  | Experimentally Observed | PTEN |
|  |  | Experimentally Observed,Moderate (predicted) | PTPRM |
|  | mmu-miR-221-5p | High (predicted) | NOTCH1 |
|  | mmu-miR-7667-3p | High (predicted) | EPN2, MYL9 |
| **Ovarian Cancer Signaling** | mmu-miR-155-5p | Experimentally Observed | CCND1, CTNNB1 |
|  |  | High (predicted) | RPS6KB1, TCF4 |
|  |  | Experimentally Observed,Moderate (predicted) | TCF7L2 |
|  | mmu-miR-7069-3p | Experimentally Observed | KRAS |
|  | mmu-miR-7069-3p | High (predicted) | PROK1, WNT8B |
|  | mmu-miR-203-3p | Experimentally Observed | ABL1, SRC |
|  | mmu-miR-222-3p | Experimentally Observed,High (predicted) | PIK3R1 |
|  | mmu-miR-222-3p | Experimentally Observed | PTEN |
|  | mmu-miR-221-5p | High (predicted) | CCND1, SUV39H1, RAD51 |
|  | mmu-miR-7667-3p | High (predicted) | BCL2, GRB2, PROK1 |
| **Osteoarthritis Pathway** | mmu-miR-155-5p | Experimentally Observed,High (predicted) | CEBPB |
|  |  | Experimentally Observed | CTNNB1, SMAD2, FADD |
|  |  | Experimentally Observed,Moderate (predicted) | SMAD1, TCF7L2 |
|  |  | High (predicted) | TCF4 |
|  | mmu-miR-7069-3p | High (predicted) | CASP7, WNT8B, LEP, PROK1 |
|  | mmu-miR-203-3p | Experimentally Observed | RUNX2 |
|  | mmu-miR-222-3p | Experimentally Observed,Moderate (predicted) | DDIT4, MMP1 |
|  |  | Experimentally Observed | FOXO3, TIMP3 |
|  | mmu-miR-221-5p | High (predicted) | CASP2, SDC4, NOTCH1, FADD |
|  | mmu-miR-7667-3p | High (predicted) | PROK1, SMAD7, SMAD9 |
| **Tec Kinase Signaling** | mmu-miR-155-5p | High (predicted) | ACTA1, VAV3, FOS |
|  |  | Experimentally Observed | FADD, RHOA, GNA13 |
|  |  | Experimentally Observed,High (predicted) | PRKCI |
|  |  | Experimentally Observed,Moderate (predicted) | TNFRSF10A |
|  | mmu-miR-7069-3p | High (predicted) | RND2 |
|  | mmu-miR-203-3p | Experimentally Observed | SRC |
|  | mmu-miR-222-3p | Experimentally Observed,High (predicted) | DIRAS3, FOS, PIK3R1 |
|  | mmu-miR-221-5p | High (predicted) | FADD, TNFRSF21, RHOD, PRKCZ, GNA14 |
|  | mmu-miR-7667-3p | High (predicted) | GNG13, GRB2 |
|  | mmu-miR-702-5p | High (predicted) | GNG13 |
| **Gα12/13 Signaling** | mmu-miR-155-5p | Experimentally Observed | CTNNB1, RHOA, GNA13, F2 |
|  |  | Experimentally Observed,Moderate (predicted) | IKBKE |
|  |  | High (predicted) | LPAR6, VAV3 |
|  | mmu-miR-7069-3p | High (predicted) | CDH20, LPAR5 |
|  |  | Experimentally Observed | KRAS |
|  | mmu-miR-203-3p | Experimentally Observed | SRC |
|  | mmu-miR-222-3p | Experimentally Observed,High (predicted) | PIK3R1 |
|  | mmu-miR-221-5p | High (predicted) | F2RL3 |
|  | mmu-miR-7667-3p | High (predicted) | GRB2, MYL9 |
| **Regulation of IL-2 in Activated and Anergic T Lymphocytes** | mmu-miR-155-5p | High (predicted) | FOS, VAV3 |
|  |  | Experimentally Observed,Moderate (predicted) | IKBKE |
|  |  | Experimentally Observed | SMAD2 |
|  | mmu-miR-7069-3p | Experimentally Observed | KRAS |
|  |  | High (predicted) | TGFB3 |
|  | mmu-miR-222-3p | Experimentally Observed,High (predicted) | FOS |
|  |  | High (predicted) | PPP3R1 |
|  | mmu-miR-221-5p | High (predicted) | CD3E |
|  | mmu-miR-7667-3p | High (predicted) | CD28, GRB2 |
|  | mmu-miR-702-5p | High (predicted) | CD247 |
| **Pancreatic Adenocarcinoma Signaling** | mmu-miR-155-5p | Experimentally Observed | CCND1, SMAD2 |
|  | mmu-miR-7069-3p | Experimentally Observed | KRAS |
|  | mmu-miR-7069-3p | High (predicted) | PROK1, TGFB3 |
|  | mmu-miR-203-3p | Experimentally Observed | ABL1 |
|  | mmu-miR-222-3p | Experimentally Observed,High (predicted) | CDKN1B, PIK3R1 |
|  |  | High (predicted) | CDKN2B |
|  | mmu-miR-221-5p | High (predicted) | CCND1, SUV39H1, RAD51, NOTCH1 |
|  | mmu-miR-7667-3p | High (predicted) | BCL2, PROK1, GRB2 |
